# Genomic Analysis Reveals a Common Breakpoint in Amplifications of the *Plasmodium vivax* Multidrug Resistance 1 Locus in Thailand

**DOI:** 10.1093/infdis/jiw323

**Published:** 2016-07-24

**Authors:** Sarah Auburn, David Serre, Richard D. Pearson, Roberto Amato, Kanlaya Sriprawat, Sheren To, Irene Handayuni, Rossarin Suwanarusk, Bruce Russell, Eleanor Drury, Jim Stalker, Olivo Miotto, Dominic P. Kwiatkowski, Francois Nosten, Ric N. Price

**Affiliations:** 1 Global and Tropical Health Division, Menzies School of Health Research, Charles Darwin University, Australia; 2 Genomic Medicine Institute, Cleveland Clinic Lerner Research institute, Ohio; 3 Wellcome Trust Sanger Institute, Hinxton; 4 Wellcome Trust Centre for Human Genetics; 5 Medical Research Council Centre for Genomics and Global Health; 6 Centre for Tropical Medicine and Global Health, Nuffield Department of Medicine, Oxford University, United Kingdom; 7 Shoklo Malaria Research Unit, Faculty of Tropical Medicine, Mahidol University, Tak; 8 Mahidol-Oxford Tropical Medicine Research Unit, Faculty of Tropical Medicine, Mahidol University, Bangkok, Thailand; 9 Department of Microbiology and Immunology, University of Otago, Dunedin, New Zealand; 10 Singapore Immunology Network, Agency for Science, Technology and Research, Singapore

**Keywords:** malaria, *Plasmodium vivax*, multidrug resistance, mdr1, copy number, mefloquine, Thailand

## Abstract

In regions of coendemicity for *Plasmodium falciparum* and *Plasmodium vivax* where mefloquine is used to treat *P. falciparum* infection, drug pressure mediated by increased copy numbers of the multidrug resistance 1 gene (*pvmdr1*) may select for mefloquine-resistant *P. vivax*. Surveillance is not undertaken routinely owing in part to methodological challenges in detection of gene amplification. Using genomic data on 88 *P. vivax* samples from western Thailand, we identified *pvmdr1* amplification in 17 isolates, all exhibiting tandem copies of a 37.6–kilobase pair region with identical breakpoints. A novel breakpoint-specific polymerase chain reaction assay was designed to detect the amplification. The assay demonstrated high sensitivity, identifying amplifications in 13 additional, polyclonal infections. Application to 132 further samples identified the common breakpoint in all years tested (2003–2015), with a decline in prevalence after 2012 corresponding to local discontinuation of mefloquine regimens. Assessment of the structure of *pvmdr1* amplification in other geographic regions will yield information about the population-specificity of the breakpoints and underlying amplification mechanisms.

Outside of Africa, *Plasmodium vivax* has become the main cause of malaria-associated morbidity [1]. Previously considered benign, it is now widely acknowledged that this species is sometimes associated with severe and life-threatening disease, particularly in young children and pregnant women [2–8]. Concerted efforts are needed to contain the spread of emerging multidrug-resistant *P. vivax* parasites [9, 10].

Although mefloquine (MQ) is not generally used for the treatment of *P. vivax* infection, indirect drug pressure in regions of *P. vivax* and *Plasmodium falciparum* coendemicity may occur where it is used in artemisinin combination therapy (ACT) to treat *P. falciparum* malaria. The high frequency of mixed-species infections [11, 12] and the high risk of *P. vivax* infection recurrence following *P. falciparum* infection [13] likely impart significant selective pressure on *P. vivax*, particularly following administration of slowly eliminated drugs, such as MQ. The molecular basis of MQ resistance in *P. falciparum* has been evaluated comprehensively, with several clinical and in vitro studies implicating copy number (CN) amplification of the *P. falciparum* multidrug resistance 1 gene (*pfmdr1*) in resistant infection [14–18]. Investigation of the genetic architecture of *pfmdr1* amplification in western Thailand highlighted the adaptive potential of *P. falciparum* parasites in response to MQ, demonstrating frequent amplification events; 5–15 independent amplification events were estimated among the isolates circulating in a single clinic [19]. Amplification of the orthologous *pvmdr1* gene has been associated with a significant decrease in the ex vivo susceptibility of *P. vivax* to MQ [20]. However, in contrast to *pfmdr1*, the genetic structure of *pvmdr1* amplification is poorly characterized. Our study sought to characterize the genetic architecture of *pvmdr1* CN variants (CNVs) in *P. vivax* isolates from western Thailand.

## MATERIALS AND METHODS

### Ethics

Ethical approval for the study was provided by Mahidol University Faculty of Medical Technology Ethics Committee (MUTM 2011-043-03) and the Oxford Tropical Research Ethics Committee (OXTREC 45-10).

### Sample Details

Samples were sourced from symptomatic patients with microscopy-confirmed monospecies *P. vivax* infection who were attending outpatient clinics in Mae Sod, western Thailand, between June 2003 and August 2015, and Timika, Papua Indonesia, between June 2004 and December 2006 (Supplementary Figure 1). MQ was not a recommended antimalarial or widely used in Papua Indonesia during the study period, and no *pvmdr1* amplifications have been reported in the region previously [20, 21]; the Indonesian isolates therefore served as controls of MQ-unexposed *P. vivax*.

### Whole-Genome Sequencing and Read Alignment

Leukocyte-depleted samples with ≥50 ng total DNA were subjected to whole-genome sequencing within the framework of a community study in the malaria Genomic Epidemiology Network [22]. Sequencing was undertaken on the Illumina GAII or Hi-Seq 2000 platform. Library preparation, cluster generation, and sequencing were undertaken as per the manufacturer's protocols for generating standard paired-end reads 75–150 base pairs long. Reads aligning to the human reference genome were removed before any analyses were undertaken. The remaining reads were aligned against the *P. vivax* SalI reference [23] (available at: http://plasmodb.org/common/downloads/release-10.0/PvivaxSal1/fasta/data/PlasmoDB-10.0_PvivaxSal1_Genome.fasta, Accessed 15 August 2016), using bwa [24], version 0.5.9-r16, with default parameters. Standard alignment metrics were generated for each sample using the bamcheck utility from samtools.

### CNV Detection and Characterization, Using Genomic Data

CNVs spanning the *pvmdr1* region (PVX_080100; Pv_Sal1_chr10:361,701–366 095) were detected using pysamstats (available at: http://github.com/alimanfoo/pysamstats, Accessed 15 August 2016). For each sample, coverage in nonoverlapping 300–base pair bins was calculated and normalized by dividing by the median coverage across all bins with the same integer percentage GC content. CNVs were called using a hidden Markov model with the python package sklearn.hmm.GaussianHMM. All CNVs >3 kilobase pairs long encompassing *pvmdr1* were recorded. Although the primary definition of genomic CN amplification was based on read depth at correctly mapping reads, assessment was also undertaken using face-away mapping reads [25].

### Multilocus Genotype (MLG) Analysis

MLG analysis was conducted using Illumina genomic data on SNPs derived from a set of 291 clinical *P. vivax* isolates [21]. ENA accession codes for all samples are available at: https://www.malariagen.net/resource/17 (Accessed 15 August 2016). Prior to analysis, samples with a genome-wide within-isolate fixation index (*F*_WS_) score of <0.95, indicative of polyclonal infection, were excluded [26, 27]. MLG analysis in the regions flanking the *pvmdr1* amplification was conducted using previously described genotype-calling definitions, whereby positions with <5 reads were assigned a missing genotype call, a minimum of 2 reference and 2 alternative alleles were required for heterozygote assignment, and all other calls were defined as homozygous for the major allele [27]. MLG analysis within the amplified region was conducted using allele frequencies derived from read counts. The relatedness between infections was illustrated for monoclonal infections with a single copy of *pvmdr1* (CN1) or multiple copies of *pvmdr1* (CN2+), using heat map plots constructed with the R heatmap.2 package.

### Single-Nucleotide Polymorphism (SNP) Genotype Calling at *pvmdr1* Y976F

The Y976F variant (Pv_Sal1_chr10:363169) did not pass the stringent criteria for inclusion in the 303K genome-wide SNP set but was included for analysis here. Genotype calls were defined as described above.

### *Pvmdr1* Amplification Breakpoint–Specific Polymerase Chain Reaction (PCR) Assay

Three PCR assays were designed to test for the presence or absence of *pvmdr1* amplification with specific breakpoints. Primer pairs MDR1LF (5′-ACTGCGAAAGTCGCCTATTT-3′) and MDR1LR (5′-TCATCGTGTGGCACATTTTT-3′), and MDR1RF (5′- GGTGAAAAGGTCGAAGCAAA-3′) and MDR1RR (5′-GGGACACGTTCCTCAGAAGT-3′) were designed as positive controls, amplifying 408–base pair and 505–base pair products, respectively, in all isolates. The test assay comprised MDR1RF and MDR1LR, which amplify an approximately 600–base pair product spanning the junction between tandem copies in multicopy (CN2+) isolates with a specific amplification breakpoint. In isolates without the tandem amplification, MDR1RF and MDR1LR are positioned in opposite orientations and so will not yield a product (Supplementary Figure 2). Each assay was undertaken in a 20-µL volume comprising 0.3 units of *Taq* DNA polymerase (Qiagen), 0.25 µM each primer, 0.2 mM dNTPs (Qiagen), 2 mM MgCl_2_, and 2 µL of genomic DNA template. Thermocycling was undertaken as follows: 95°C for 3 minutes, followed by 35 cycles of 95°C for 30 seconds, 58°C for 40 seconds, and 72°C for 30 seconds, with a final step of 72°C for 5 minutes. The PCR products were assessed by standard agarose gel electrophoresis.

### Quantitative PCR Data on *pvmdr1*

Details on *pvmdr1* CN determined by SYBR Green–based quantitative PCR (qPCR) were sourced from a previous study, whereby CN amplification was defined as ≥1.5 ratio of *pvmdr1* amplification against β-tubulin [20]. DNA was available for 48 Thai and 27 Indonesian isolates from the study.

### STR Genotyping to Determine Multiplicity of Infection

Genotyping was undertaken at 3 short-tandem-repeat (STR) markers—*Pv3.27, msp1F3*, and *MS16*—that have demonstrated high genetic diversity in previous studies [28–33]. Amplification was undertaken using previously described methods [29]. The labeled PCR products were sized by denaturing capillary electrophoresis on an ABI 3100 Genetic Analyzer. Genotype calling was facilitated with GeneMapper, version 4.0. A minimum of 1 multiallelic locus was required to define an infection as polyclonal, and a minimum of 3 monoallelic loci were required to define an infection as monoclonal; all other infections were classified as genotype fails.

### Statistical Tests

Proportions were examined using the Fisher exact test. Spearman rank correlation was used to test correlations in nonparametric data. All tests were performed using R software, version 2.12.1, and assuming a significance threshold of 0.05.

## RESULTS

### *pvmdr1* CN Amplification Identified Using Genomic data

**Figure 1.**
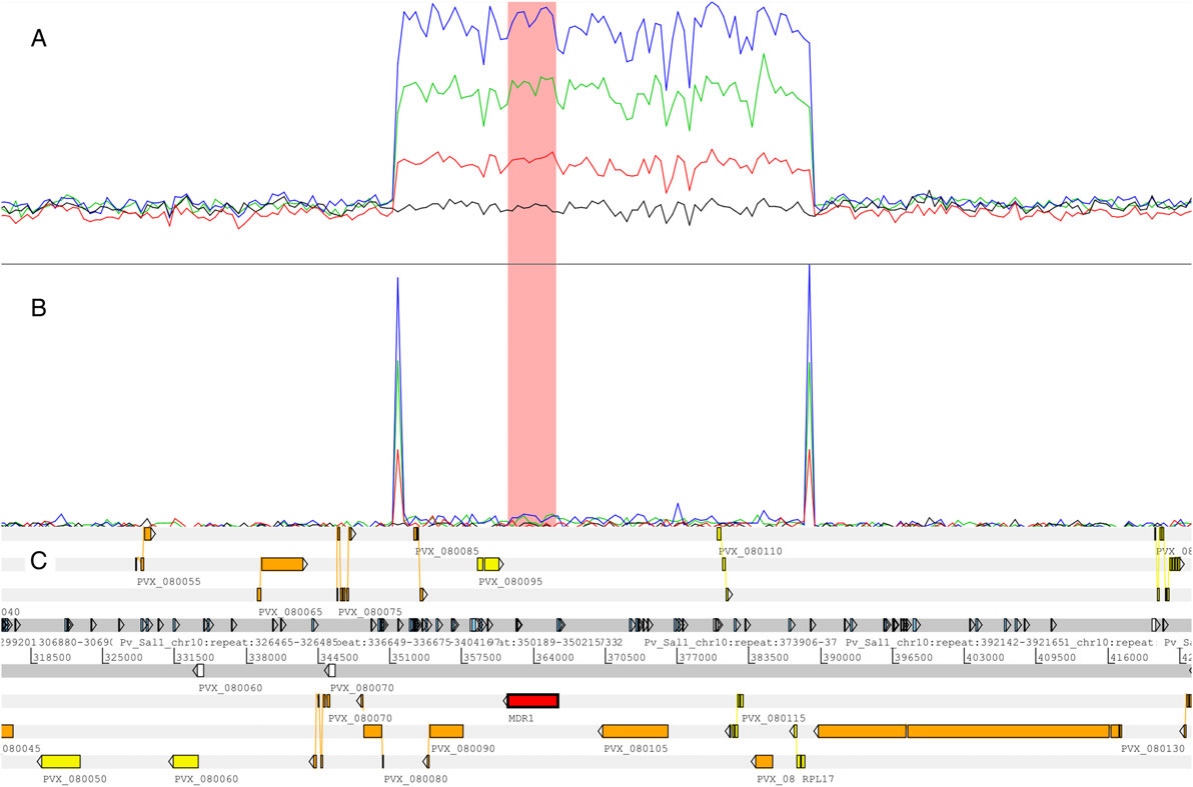
Illustration of the breakpoint region and read depth variation in examples of isolates with no amplification and with 2, 3, and 4 predicted copies. *A*, Depth (height) of correctly mapping reads in examples of copy number (CN) 1 (black), CN2 (red), CN3 (green), and CN4 (blue) clonal isolates. *B*, Depth (height) of face-away mapping reads in the same samples, illustrating the breakpoint region. *C*, Genomic features in the region, including repeat regions. The *pvmdr1* gene region is highlighted in pink in panels *A* and *B*.

High-read-depth genomic data were available for 88 Thai *P. vivax* isolates. The median read depth across the 21.4–megabase pair core genome was 67.5 (range, 16–111) in these isolates. Seventeen isolates (19.3%) demonstrated increased read depth and face-away read profiles reflective of CN amplification in the region encompassing *pvmdr1* (Supplementary Figure 3). Three additional infections (PD0173-C, PD0183-C, and PD0614-C) demonstrated evidence of amplification by face-away read mapping but not by read depth (Supplementary Figure 3). The majority of the amplified samples (14 [82%]) appeared to have 2 copies of the *pvmdr1* region, whereas 2 (12%) had 3 copies, and 1 (6%) had 4 copies. Figure 1 illustrates the read coverage profiles of correctly mapping and face-away mapping reads in the region in each of a CN1, CN2, CN3, and CN4 isolate. Visual inspection of the alignments revealed a sequence of soft-clipped bases (ie, regions of the read that do not align in the same genomic region as the rest of the read) present on both the 5′ and 3′ ends of the amplified region, denoting the amplification breakpoints (Supplementary Figure 4). Common 5′ and 3′ breakpoint sequences consisting of 15–base pair and 18–base pair poly-A tracts, starting at Pv_Sal1_chr10:351600 and Pv_Sal1_chr10:389205, respectively, were observed in all CN2+ samples. In addition to *pvmdr1*, the 37.6–kilobase pair amplified region encompassed genes encoding 3 hypothetical proteins (PVX_080085, PVX_080105, and PVX_080120), a mitochondrial processing peptidase α protein (PVX_080095), a putative G10 protein (PVX_080110), a putative iron-sulfur cluster assembly accessory protein (PVX_080115), and a putative 50S ribosomal protein L17 (PVX_080125; Figure 1).

### MLG Patterns in the Regions Flanking the *pvmdr1* CNV

Fifty-six of 88 isolates (63.6%), including 10 of 17 CN2+ isolates (58.8%), exhibited a genome-wide *F*_WS_ of ≥ 0.95, consistent with monoclonal infections, and were included in MLG analysis. A total of 105 and 136 SNPs within 20 kilobase pairs of the 5′ and 3′ flanking regions, respectively, of the amplified region had at least 1 minor variant among the high-*F*_WS_ samples. As illustrated in Supplementary Figure 5, a broad range of MLGs were observed, and there was no notable clustering of the CN2+ samples in either the 5′ or 3′ flanking regions.

### MLG Patterns Within the *pvmdr1* CNV Region

**Figure 2.**
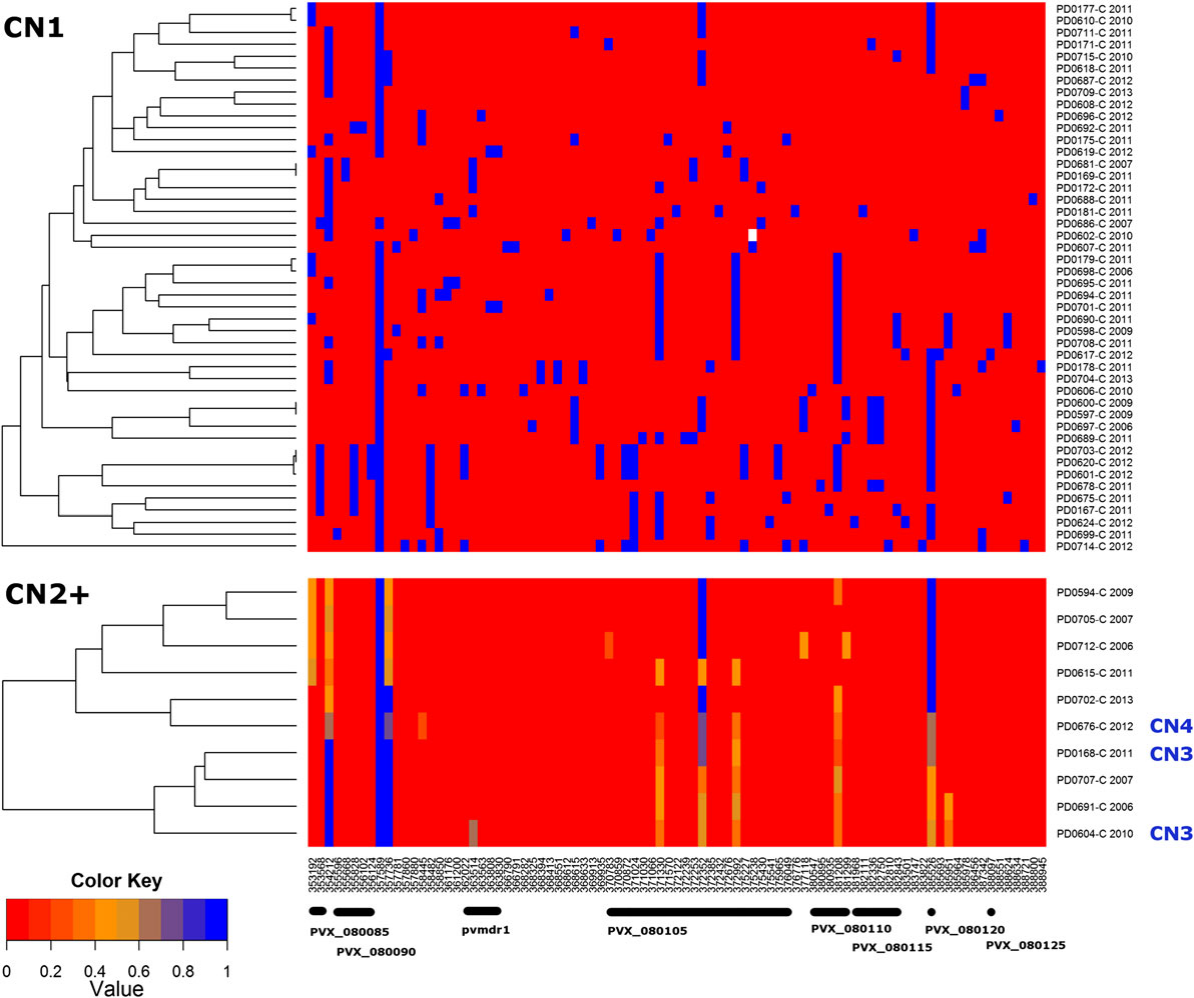
Heat map illustrating the relatedness of multilocus genotypes (MLGs) in the *pvmdr1* region in CN1 (top) and CN2+ (bottom) isolates. MLGs were reconstructed using allele frequency data at 87 single-nucleotide polymorphisms in the amplified region. Color is used to represent the nonreference allele frequency (proportion of reads containing the nonreference allele within that sample). Homozygote reference (Sal1) alleles are presented in red (0.0) and alternative alleles in blue (1.0). Missing genotype calls are colored white. Copy number (CN) definitions are based on genomic read depth results. Samples with a *F*_WS_ of < 0.95 have been excluded. MLGs are labeled with a sample identifier and year of collection.

Eighty-seven SNPs within the 37.6–kilobase pair amplified region had at least 1 minor variant among the high-*F*_WS_ samples. With the exception of 1 genotype fail in 1 sample, all positions were successfully called. As illustrated in Figure 2, the large majority of positions in the CN1 isolates were homozygote for the reference or alternative allele, as expected in monoclonal samples. A broad range of MLGs were observed, with few isolates sharing identical or near-identical MLGs. The MLGs in the CN2+ isolates were more complex to interpret owing to multiple apparent heterozygote positions. Given the stringent *F*_WS_ filtration to remove polyclonal infections, the heterozygote positions likely reflected allelic differences between copies. In accordance with this hypothesis, the median MAF of the heterozygote genotypes approximated the expected values of 50% (range, 42%–49%) in the 7 CN2 isolates, 33% in the 2 CN3 isolates (36% and 35%), and 25% in the CN4 isolate (29%; Supplementary Table 1). A total of 14 loci displayed at least 1 heterozygote genotype. The minor variants at these loci were not private to the CN2+ group; all 14 loci segregated among the CN1 samples. Furthermore, there was a significant correlation between the allele frequency at these loci in the CN1 vs CN2+ samples (ρ = 0.66, *P* = .011). These patterns suggest ongoing genetic exchange between CN1 and CN2+ isolates. The heterozygote positions clustered at the 5′ and 3′ ends of the amplified region, with *pvmdr1* and the direct flanking sequence remaining largely invariable in the CN2+ samples, possibly reflecting recombination sites at the 5′ and 3′ ends of *pvmdr1* (Figure 2). On visual inspection of the MLGs, we identified several combinations of CN1 MLGs that would produce heterozygote patterns observed at the 5′ or 3′ ends of *pvmdr1* in CN2+ isolates; 2 examples are illustrated in Supplementary Figure 6.

### Relationship Between *pvmdr1* Duplication and Y976F Variation

Genotype calls were determined for the Y976F locus in the 88 isolates with genomic data. Among the 17 CN2+ isolates, the majority (94%) displayed the homozygote wild-type genotype (Supplementary Table 2). None (0%) of the CN2+ isolates exhibited a homozygous mutant genotype, compared with 11 of 71 CN1 isolates (15.5%), but the difference was not significant (*P* > .05).

### Validation of a *pvmdr1* Amplification Breakpoint–Specific PCR Assay

The presence of a common breakpoint enabled the design of a targeted PCR assay to detect CN2+ isolates in samples of varying quality, including those not currently amenable to whole-genome sequencing. No amplification was observed in human and *P. falciparum* DNA. Among the 88 isolates with genomic data, 1 isolate (1%) with CN1 based on read depth was not defined as CN1 the PCR assay. All 17 isolates with CN2+ based on read depth were confirmed by the breakpoint-specific assay. Among the 71 infections with a CN1 read depth status, 13 (18.3%), including PD0173-C, PD0183-C, and PD0614-C, demonstrated evidence of a subpopulation of CN2+ clone(s), (30 CN2+ isolates [34.1%] in total; Table 1). STR-based analysis revealed that all 13 infections (100%) were polyclonal.

**Table 1.** Concordance Between the Breakpoint-Specific PCR vs Read Depth and qPCR in Detecting pvmdr1 CN Variation

Variable	Breakpoint-Specific PCR				Concordance, %
	CN1	CN2+	Fail	Total	
Sequence read depth: Thailand					
CN1	57 (22^a^)	13 (13)	1	71	…
CN2+	0	17 (7)	0	17	…
Total	57	30	1	88	85
qPCR: Thailand					
CN1	33 (11^b^)	3 (2)	1	37	…
CN2+	1 (0)	9 (4)	1	11	…
Total	34	12	2	48	91
qPCR: Indonesia					
CN1	25	0	2	27	…
CN2+	0	0	0	0	…
Total	25	0	2	27	100

Number of polyclonal infections as defined by microsatellite genotyping are indicated in parentheses. Genotyping was not undertaken in the Indonesian isolates.

Abbreviation: CN, copy number.

^a^ Two isolates with genotyping failure.

^b^ One isolate with genotyping failure.

DNA was available for 48 Thai isolates with quantitative PCR (qPCR) data on *pvmdr1* CN from a previous study [20]. Forty-six isolates (95.8%) yielded confident results with the breakpoint-specific assay. Ten of these isolates (21.7%) demonstrated evidence of increased *pvmdr1* amplification, of which 9 (90%) were confirmed with the breakpoint-specific assay (Table 1). Among the 36 isolates defined as CN1 by qPCR, 3 (8%) demonstrated evidence of carrying at least 1 CN2+ clone (12 CN2+ isolates [26.1%] in total). Two of the 3 infections revealed evidence of polyclonality with STR analysis, versus 4 of 9 isolates with CN2+ status detected by both methods. In 27 Indonesian isolates with qPCR data and DNA available, 25 (93%) yielded confident results with the breakpoint-specific assay. No evidence (0%) of *pvmdr1* amplification was observed by qPCR or with the breakpoint-specific assay (Table 1).

### Temporal Trends in the Prevalence of *pvmdr1* Duplication in Thailand Between 2003 and 2015

**Figure 3.**
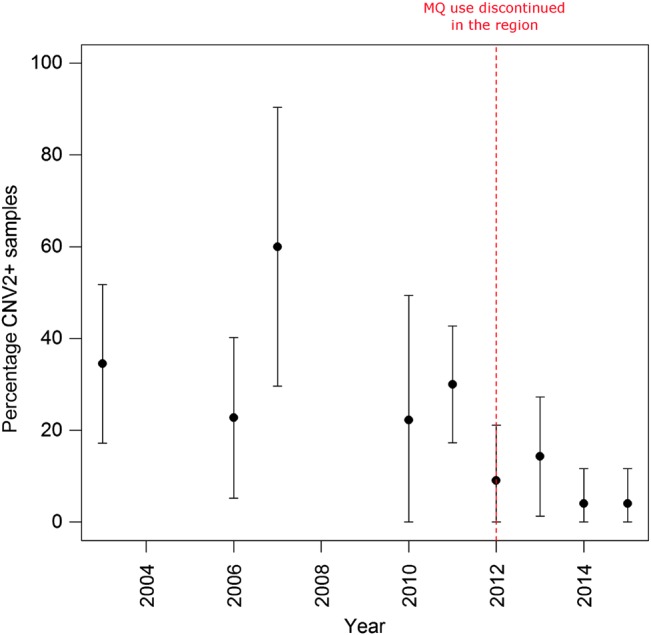
Temporal trend in the prevalence of *pvmdr1* amplification. Data from 2008 and 2009 were excluded owing to low sample size (1 and 4, respectively). Abbreviation: MQ, mefloquine.

The breakpoint-specific assay was attempted on 226 Thai samples, including the genomic and qPCR samples. A total of 220 isolates (95.2%) yielded successful assays; 215 of these isolates had details on the date of blood sampling. Sampling dates ranged from 2003 to 2015, although sample size was low in 2008 (n = 1) and 2009 (n = 4) (Supplementary Table 3). As illustrated in Figure 3, with the exclusion of 2008–2009, a trend for declining prevalence of *pvmdr1* CN2+ clones was observed over time. A significant difference was observed in CN2+ prevalence in the years before (2003–2011 [52%]) vs after (2012–2015 [17%]) MQ regimens were discontinued in the region (*P* = 1.635 × 10^–5^).

## DISCUSSION

This study provides the first in-depth description of the genetic architecture of *pvmdr1* CN amplification. In contrast to the dynamics of *pfmdr1* amplification in western Thailand, we observed the same *pvmdr1* amplification breakpoints over a 12-year period. Capitalizing on the invariability of the breakpoint, we describe the application of a simple breakpoint-specific PCR assay to temporal surveillance of *pvmdr1* amplification in western Thailand.

The simplest explanation for the conservation of the *pvmdr1* amplification breakpoint is that the amplifications have all descended from a single ancestral event. Given that isolates with 3 and 4 copies of the *pvmdr1* region were observed, it seems there has been >1 amplification event, but the conservation of the breakpoint in these CN3+ isolates suggests that additional amplifications may have taken place on the background of a common ancestral duplication. One might expect to observe comparably high genetic relatedness in the region flanking the amplification in CN2+ versus CN1 isolates if the former have a recent common ancestor. MLG analysis in the regions flanking the amplification did not infer a single lineage of the CN2+ isolates. However, one cannot exclude the possibility that the genetic relatedness between the CN2+ isolates has been broken down by recombination with CN1 parasites over time. Indeed, de-amplification events might further obscure detection of a common lineage. Although the date at which a founding amplification event might have taken place remains unclear, we observed evidence of the same breakpoint in isolates from 2003—the earliest year tested.

The assumption of a single origin of the common breakpoint contrasts with the dynamics of *pfmdr1* amplification in the same geographic region. A range of *pfmdr1* breakpoints and amplicon sizes were observed in *P. falciparum* isolates collected from a single clinic between 2000 and 2003 [19]. Indeed, laboratory experiments infer high rates of *pfmdr1* duplication (1 per 10^8^ parasites) in drug selection experiments [34]. However, although *P. vivax* may be exposed to MQ in mixed-species infections, misdiagnoses, and recurrences following *P. falciparum* infection, the selective pressure of this concomitant drug exposure is not likely to be as strong as that for *P. falciparum*, possibly resulting in fewer founding amplification events. Differences in transmission dynamics between the 2 species are also likely to contribute to the contrasting *pfmdr1 and pvmdr1* amplification patterns. The early development of the transmissible gametocyte stages in *P. vivax* may have further reduced the impact of drug selection [35]. In addition, the ability of *P. vivax* isolates to form dormant liver stages, which relapse weeks or months after inoculation, may facilitate longer persistence of certain lineages in *P. vivax* versus *P. falciparum*.

Although the degree of selective pressure on *P. vivax* parasites remains unclear, a recent study identified frequent duplications in the *P. vivax* Duffy binding protein 1 gene (*pvdbp1*) in Madagascar, which also had common breakpoints [25]. Sequence analysis inferred a single and recent ancestral origin of the *pvdbp1* duplication in these isolates. The authors identified the same *pvdbp1* breakpoints in non-Malagasy *P. vivax* infections but could not determine whether these isolates had a shared lineage with the Malagasy isolates. In combination with our results on *pvmdr1*, these findings suggest that common amplification breakpoints may not be unusual in *P. vivax*.

The possibility that multiple independent *pvmdr1* amplification events have occurred using the same breakpoints cannot be excluded. It remains unclear, however, why the same breakpoints would be used, indeed in contrast to *P. falciparum*, where multiple breakpoints are evident [19]. The breakpoints of *pfmdr1* and *P. falciparum* GTP-cyclohydrolase 1 amplifications contain low-complexity sequences such as monomeric A/T tracts [19, 36, 37]. Similarly, the breakpoints of the common *pvdbp1* and *pvmdr1* duplications contained long homopolymer tracts of T and A residues, respectively [25]. Although several repetitive sequences are observed in the regions flanking *pvmdr1*, it is possible that the DNA conformation or other mechanism(s) lead to preferential breakage at the poly-A tracts. Alternatively, amplifications may have occurred at other breakpoints but were associated with a fitness disadvantage, compared with those described in the current study, and thus have not reached high frequency.

We observed evidence of different allelic states between copies in isolates with *pvmdr1* amplifications, as inferred by the presence of heterozygote calls in single-clone infections. Assuming that the mechanism of the original amplification event entailed duplication during mitosis, the ancestral duplication would have comprised identical copies and thus exhibited no heterozygote states. Over time, mutation and/or recombination events may have created different allelic states between copies in some lineages. Indeed, none of the polymorphisms among the CN2+ isolates were private to this group; rather, they were also variable in the CN1 group, possibly inferring occasional recombination between CN1 and CN2+ isolates. Alternatively, the original amplification(s) may have been produced during meiosis, with distinct maternal and paternal copies being retained. In this scenario, the ancestral amplification(s) could carry copies with different allelic states.

Similar to *P. falciparum*, ex vivo investigation infers that, although *pvmdr1* amplification reduces susceptibility to MQ, it increases susceptibility to chloroquine (CQ) [20]. Although requiring further confirmation, a point mutation conferring a Y976F change in *pvmdr1* has been associated with low-level reduction in CQ susceptibility [38]. We were able to assess both *pvmdr1* amplification and 976 variation in 88 isolates collected between 2006 and 2013. In accordance with a previous study undertaken in western Thailand [39], although 976-F variants were observed in our study, they were not observed in amplifications. This trend may reflect a significant fitness cost to the parasite of carrying both the mutation and amplification and lends suggestion that MQ pressure on the *P. vivax* population was possibly greater than CQ pressure during the sampling period.

Quantitative methods for detecting amplifications, such as read depth and quantitative PCR approaches, have the benefit that they are not constrained to specific amplicons. However, these approaches have limited sensitivity to detect low-frequency CN2+ clones in polyclonal infections—a caveat overcome by the breakpoint-specific PCR approach. In regions where polyclonal infections are common, quantitative approaches may miss a sizeable proportion of CN2+ clones. Indeed, relative to the breakpoint-specific PCR, 43% and 25% of infections with *pvmdr1* amplification were missed by read depth (13 of 30) and qPCR approaches (3 of 12), respectively, in our study. The sensitivity, simplicity, and applicability to a range of sample qualities were advantageous features of the breakpoint-specific assay for surveillance of *pvmdr1* amplification in western Thailand. Using this assay, we demonstrated significantly lower prevalence of CN2+ clones between 2012 and 2015 relative to earlier years tested. This trend is consistent with the local discontinuation of MQ plus artesunate in 2012 following documentation of its declining efficacy in the region [40]. However, the application of any assay using a specific breakpoint pattern is critically reliant on knowledge of the genetic background of the CN2+ clones circulating in the given population. Further investigation of the genetic background of *pvmdr1* amplification is required in other geographic regions. These investigations will provide further insights into the evolutionary dynamics of *pvmdr1* amplification and its adaptive potential.

In contrast to *pfmdr1*, the breakpoints of *pvmdr1* CN amplification appear to be highly conserved in western Thailand. It remains unclear whether this dynamic reflects a single amplification origin and whether the same breakpoints are commonly observed in other geographic regions, permitting surveillance of reduced MQ susceptibility by using a rapid and cost-effective breakpoint-specific PCR approach.

## Supplementary Data

Supplementary materials are available at http://jid.oxfordjournals.org. Consisting of data provided by the author to benefit the reader, the posted materials are not copyedited and are the sole responsibility of the author, so questions or comments should be addressed to the author.

Supplementary Data
