# Supplementary material for: Genomic Analysis Reveals a Common Breakpoint in Amplifications of the Plasmodium vivax Multidrug Resistance 1 Locus in Thailand
Source: J Infect Dis. 2016 Jul 24;214(8):1235–42. doi: 10.1093/infdis/jiw323 (PMC5034950; doi:10.1093/infdis/jiw323)
Supplement: Supplementary Data [file supp_214_8_1235__index.html]

Genomic Analysis Reveals a Common Breakpoint in Amplifications of the Plasmodium vivax Multidrug Resistance 1 Locus in Thailand — Genomic Analysis Reveals a Common Breakpoint in Amplifications of the Plasmodium vivax Multidrug Resistance 1 Locus in Thailand — Supplementary Data 

# Genomic Analysis Reveals a Common Breakpoint in Amplifications of the *Plasmodium vivax* Multidrug Resistance 1 Locus in Thailand

## Supplementary Data

Supplementary Data

- Supplementary Data - Pdf file
