# Supplementary material for: Genomic Analysis Reveals a Common Breakpoint in Amplifications of the Plasmodium vivax Multidrug Resistance 1 Locus in Thailand
Source: J Infect Dis. 2016 Jul 24;214(8):1235–42. doi: 10.1093/infdis/jiw323 (PMC5034950; doi:10.1093/infdis/jiw323)
Supplement: Supplementary Data [file supp_jiw323_jiw323supp.pdf]

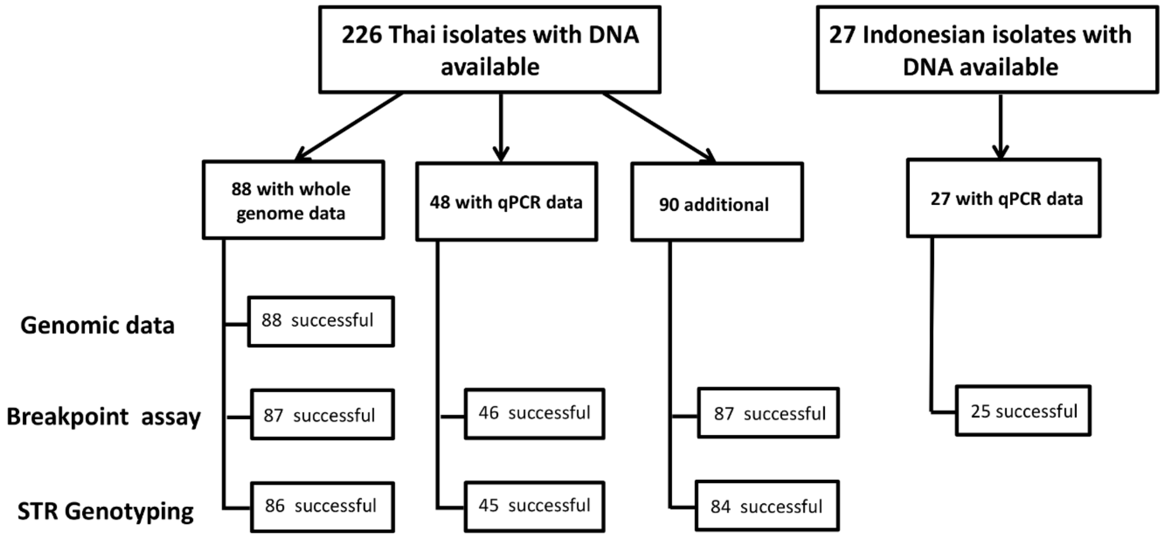

Supplementary Figure 1. Flow chart of samples used in the study.

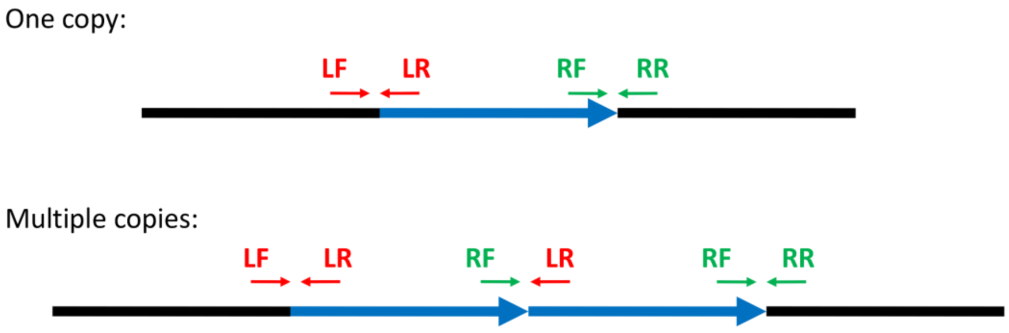

Supplementary Figure 2. Schematic of the *pvmdr1* breakpoint assay.

Diagram of the *pvmdr1* duplication and orientation of PCR primers to amplify control upstream (LF+LR; F = forward, R = reverse) and control downstream (RF+RR) fragments. The RF+LR primer combination will only produce a PCR amplicon if the *pvmdr1* region is duplicated in tandem.

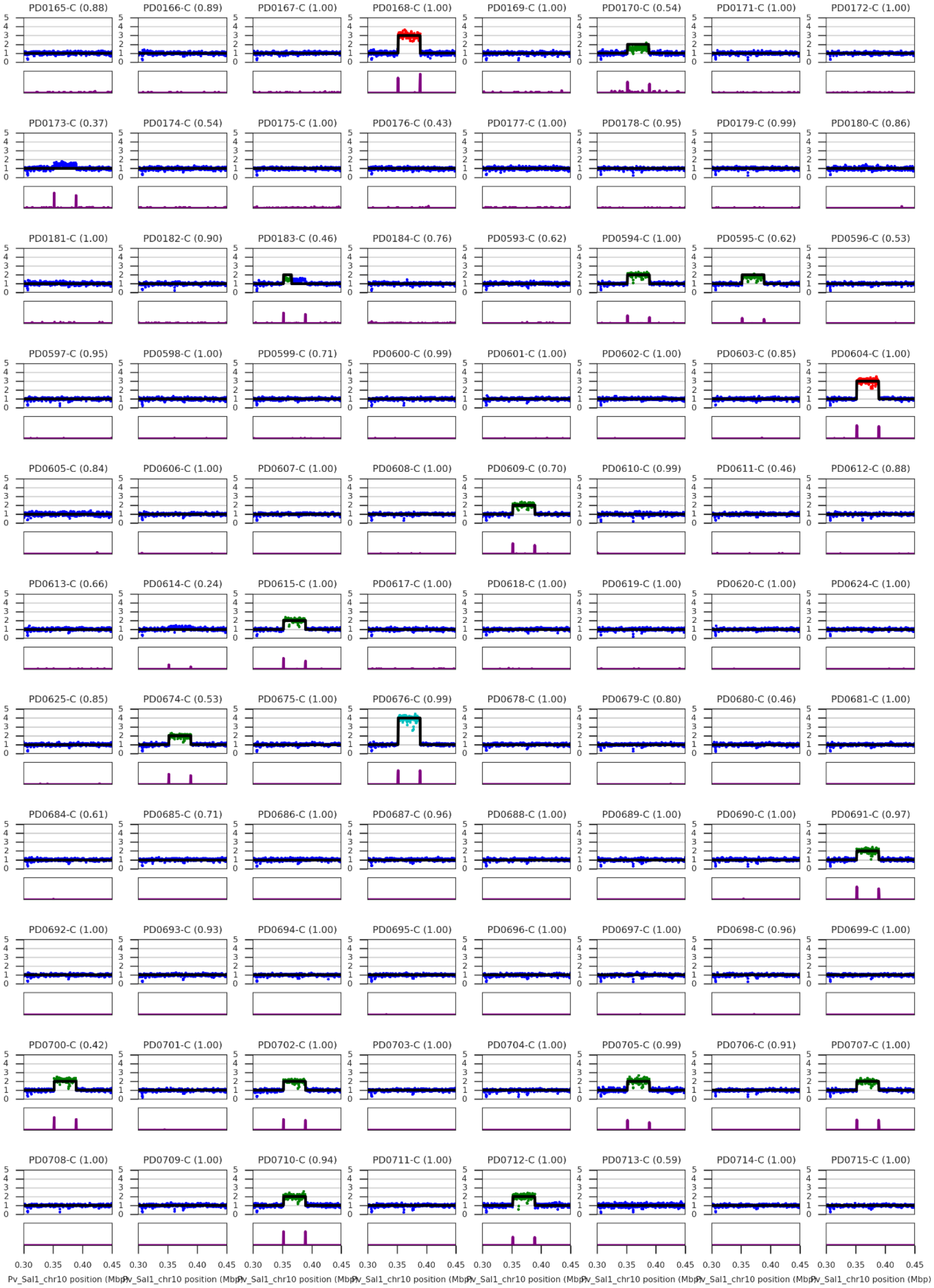

Supplementary Figure 3. Illumina read depth CN patterns in the *pvmdr1* region in all 88 Thai isolates.

In each plot, the upper panel illustrates the estimated number of copies (y-axis) at the genomic positions indicated on the x-axis as determined using correctly mapping reads. Positions are colour-coded by estimated number of copies; blue = 1, green = 2, red = 3 and turquoise = 4. The lower panel illustrates the depth of faceaway reads, highlighting the breakpoints. The F_{WS} score is provided in brackets adjacent to the sample ID in the plot label.

A. Left breakpoint

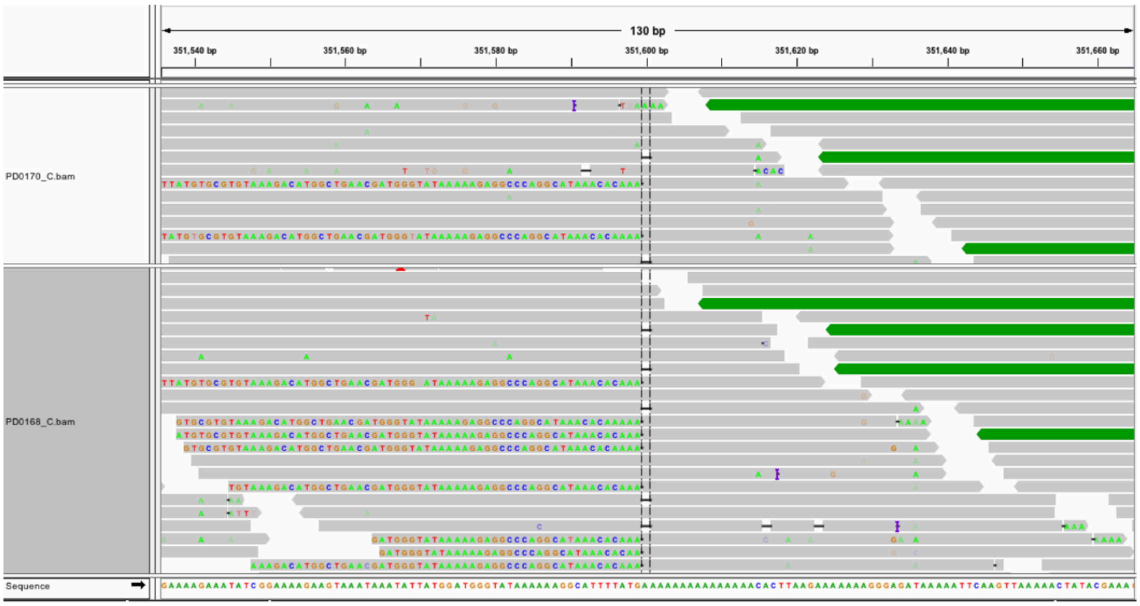

B. Right breakpoint

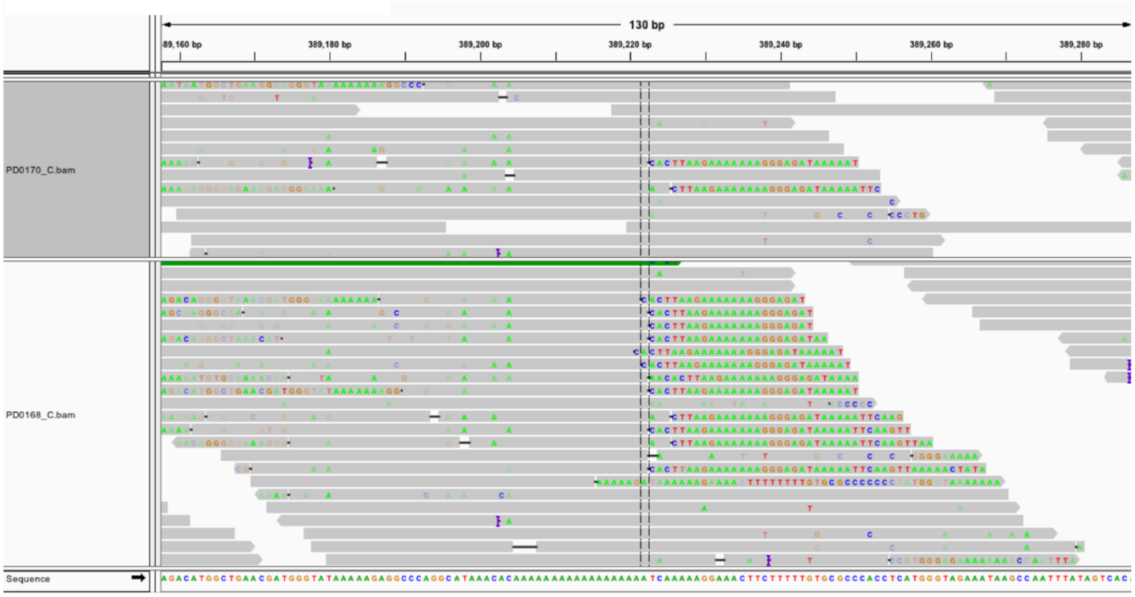

Supplementary Figure 4. Illustration of the soft-clipped bases at the breakpoint regions.

IGV (Integrative Genomics Viewer) screenshots of the read alignments at the left (5’) (panel A) and right (3’) (panel B) breakpoint regions in isolates with multiple copies. Nucleotides presented in colour reflect soft-clipped bases i.e. regions of the read that do not align in the same genomic region as the rest of the read. At the left breakpoint (panel A), the soft-clipped sequence (AAAAAGAGGCCCAGGCATAAACACAAA) exactly matches the reference sequence 5’ of the right breakpoint. Likewise, the soft-clipped portion of the reads at the left breakpoint (CACTTAAGAAAAAAAGGGAGAT) exactly matches the reference sequence 3’ of the left breakpoint.

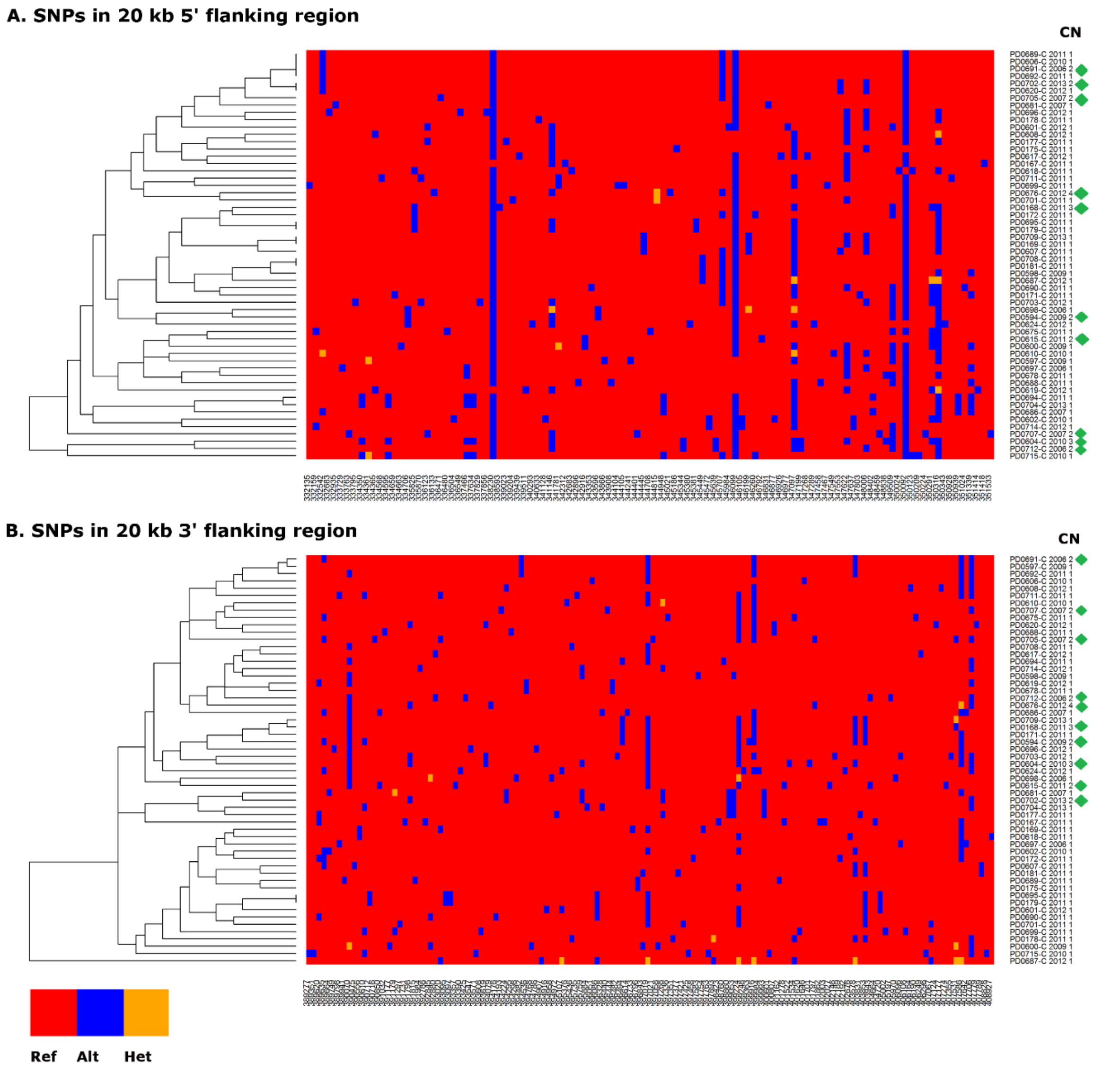

Supplementary Figure 5. Heatmap illustrating the relatedness of MLGs in the regions flanking the *pvmdr1* CNV. Multi-locus genotypes (MLGs) were reconstructed using genotype calls (homozygote

reference in red, homozygote alternative in blue, heterozygote in orange) at 105 and 136 SNPs in the 20 kb regions 5' and 3' respectively of the *pvmdr1* copy number variation (CNV). Copy number (CN) definitions are based on genomic read depth results. Samples with F_{WS}<0.95 have been excluded. MLGs are labeled with sample ID, year of collection and CN. CN2+ isolates are highlighted with green diamonds.

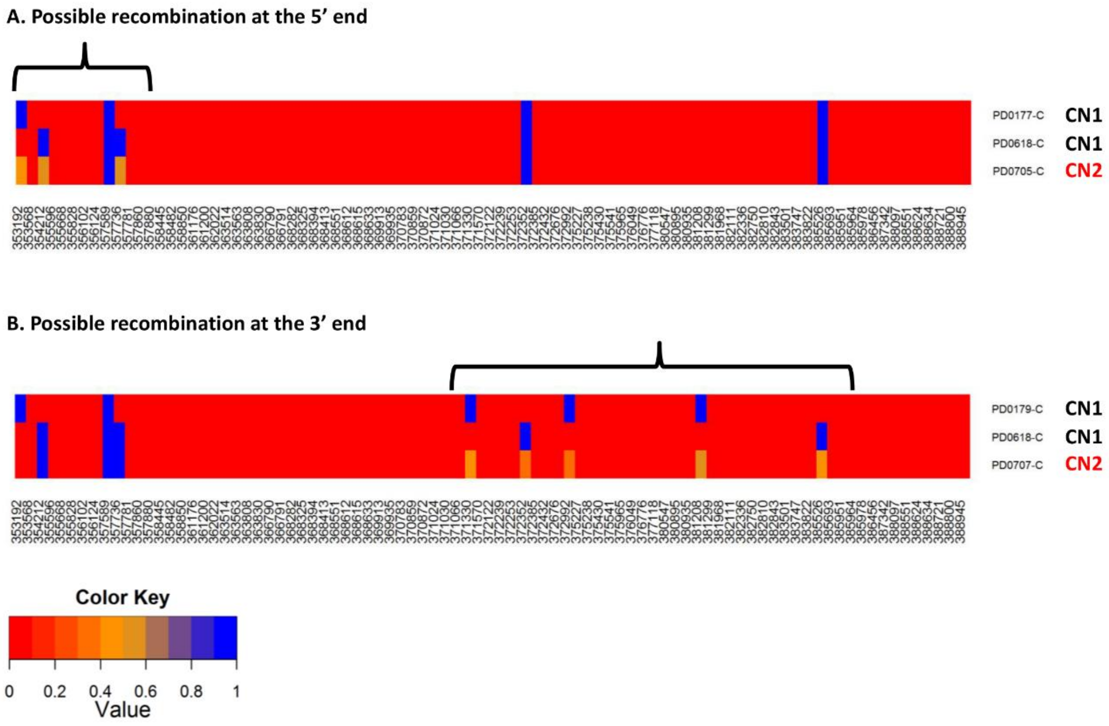

Supplementary Figure 6. Examples of potential recombination events generating different allele states between copies.

Multi-locus genotypes (MLGs) were reconstructed using allele frequency data at 87 Single Nucleotide Polymorphisms in the amplified region. Homozygote reference (Sal1) alleles are presented in red (0.0) and alternative alleles in blue (1.0). Copy number (CN) definitions are based on genomic read depth results. Samples with F_{WS}<0.95 have been excluded.

Sample	No. copies	No. heterozygote positions	Median read depth (range)	Median MAF at heterozygote positions
PD0594-C	2	4	192 (184-220)	0.43
PD0615-C	2	6	259 (243-296)	0.44
PD0691-C	2	6	118.5 (97-154)	0.42
PD0702-C	2	2	179 (172-186)	0.49
PD0705-C	2	3	78 (69-85)	0.45
PD0707-C	2	5	165 (154-186)	0.47
PD0712-C	2	6	106.5 (84-129)	0.43
PD0168-C	3	5	74 (65-93)	0.36
PD0604-C	3	7	291 (258-323)	0.35
PD0676-C	4	8	412.5 (393-470)	0.29

Supplementary Table 1. Minor allele frequency of heterozygote positions within the amplified region in CN2+ samples.

MAF = minor allele frequency

CN status	Homozygote wild-type	Homozygote mutant	Heterozygote
**CN1**	53 (74.6%)	11 (15.5%)	7 (9.9%)
**CN2+**	16 (94.1%)	0 (0%)	1 (5.9%)

Supplementary Table 2. Relationship between *pvmdr1* Y976F genotype status and copy number variation in Thailand. Thai genomic dataset (*n* = 88). CN = copy number. Percentage of CN1 or CN2+ isolates with given genotype are presented in parentheses. CN status was determined by read depth.

Collection year	No. samples	CN1	CN2+ (%)
2003	29	19	10 (34.5%)
2006	22	17	5 (22.7%)
2007	10	4	6 (60%)
2008	1	0	1 (100%)
2009	4	3	1 (25%)
2010	9	7	2 (22.2%)
2011	50	35	15 (30%)
2012	22	20	2 (9.1%)
2013	28	24	4 (14.3%)
2014	25	24	1 (4%)
2015	25	24	1 (4%)

Supplementary Table 2. Prevalence of *pvmdr1* amplifications in western Thailand between 2003 and 2015.
